# Functional Implications of Species Differences in the Size and Morphology of the Isthmo Optic Nucleus (ION) in Birds

**DOI:** 10.1371/journal.pone.0037816

**Published:** 2012-05-29

**Authors:** Cristián Gutiérrez-Ibáñez, Andrew N. Iwaniuk, Thomas J. Lisney, Macarena Faunes, Gonzalo J. Marín, Douglas R. Wylie

**Affiliations:** 1 University Centre for Neuroscience, University of Alberta, Edmonton, Alberta, Canada; 2 Department of Neuroscience, Canadian Centre for Behavioural Neuroscience, University of Lethbridge, Lethbridge, Alberta, Canada; 3 Facultad de Ciencias, Departamento de Biología, Universidad de Chile, Nunoa, Santiago, Chile; 4 Department of Psychology, University of Alberta, Edmonton, Alberta, Canada; 5 Facultad de Medicina, Universidad Finis Terrae, Providencia, Santiago, Chile; Claremont Colleges, United States of America

## Abstract

In birds, there is a retinofugal projection from the brain to the retina originating from the isthmo optic nucleus (ION) in the midbrain. Despite a large number of anatomical, physiological and histochemical studies, the function of this retinofugal system remains unclear. Several functions have been proposed including: gaze stabilization, pecking behavior, dark adaptation, shifting attention, and detection of aerial predators. This nucleus varies in size and organization among some species, but the relative size and morphology of the ION has not been systematically studied. Here, we present a comparison of the relative size and morphology of the ION in 81 species of birds, representing 17 different orders. Our results show that several orders of birds, besides those previously reported, have a large, well-organized ION, including: hummingbirds, woodpeckers, coots and allies, and kingfishers. At the other end of the spectrum, parrots, herons, waterfowl, owls and diurnal raptors have relatively small ION volumes. ION also appears to be absent or unrecognizable is several taxa, including one of the basal avian groups, the tinamous, which suggests that the ION may have evolved only in the more modern group of birds, Neognathae. Finally, we demonstrate that evolutionary changes in the relative size and the cytoarchitectonic organization of ION have occurred largely independent of phylogeny. The large relative size of the ION in orders with very different lifestyles and feeding behaviors suggest there is no clear association with pecking behavior or predator detection. Instead, our results suggest that the ION is more complex and enlarged in birds that have eyes that are emmetropic in some parts of the visual field and myopic in others. We therefore posit that the ION is involved in switching attention between two parts of the retina i.e. from an emmetropic to a myopic part of the retina.

## Introduction

**Figure 1:**
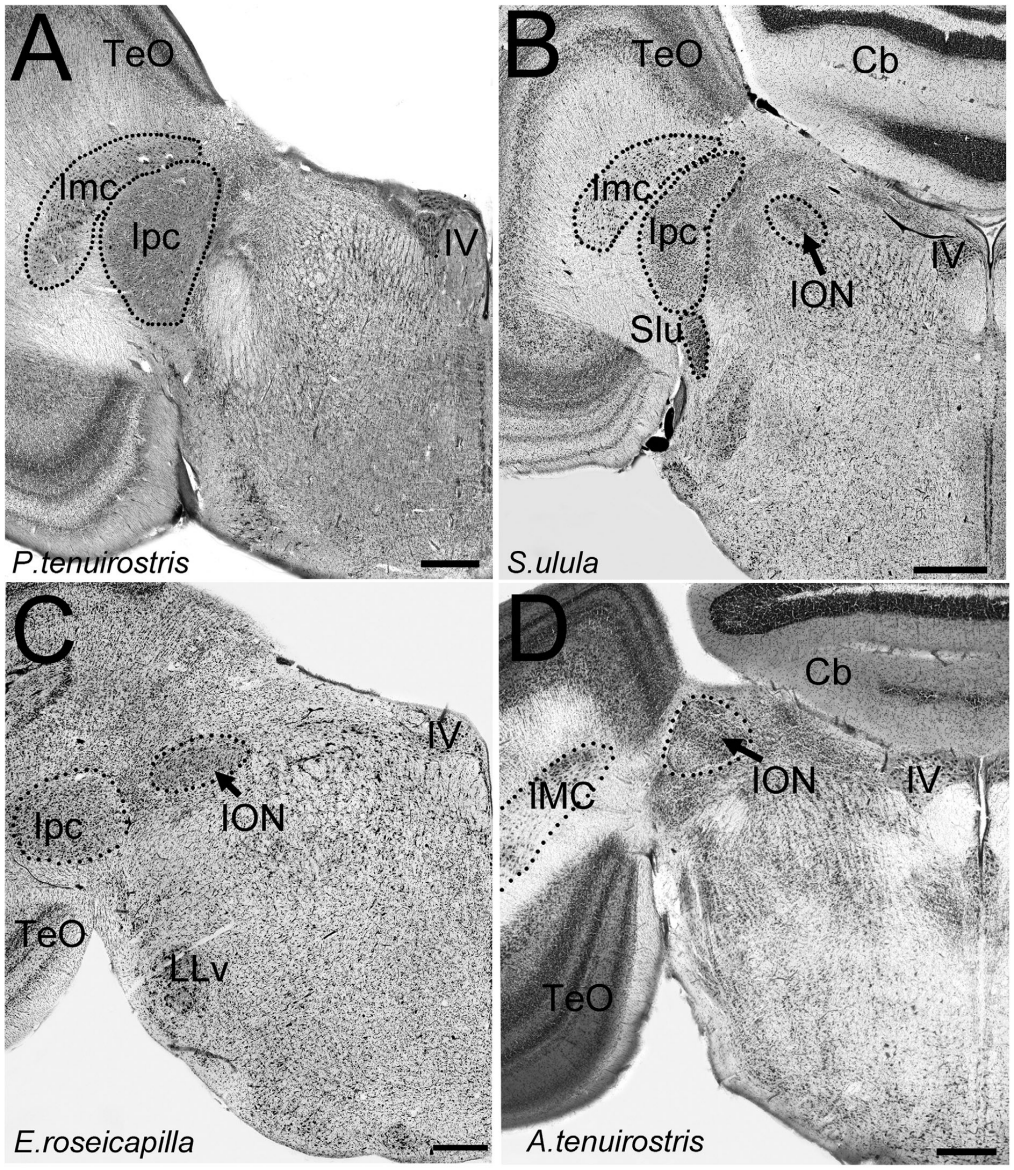
Location of the isthmo optic nucleus (ION) in the brainstem. Photomicrographs of coronal sections through the brainstem of different species of birds showing the location of the isthmo optic nucleus (ION). **A** shows the absence of ION in a seabird (Procellariiformes), the Short-tailed Shearwater (*Puffinus tenuirostris*). The coronal section is through the brainstem, at the level of the trochlear nucleus (**IV**), where the ION is usually found in other birds. **B** to **D** show the ION in (**B)** an owl (Strigiformes), the Northern Hawk Owl (*Surnia ulula*); (**C)** a parrot (Psittaciformes), the Galah (*Eolophus roseicapilla*); (**D)** a songbird (Passeriformes), the Eastern Spinebill (*Acanthorhynchus tenuirostris*). **Imc**  =  nucleus isthmi magnocellularis; **Ipc**  =  nucleus isthmi parvocellularis; **TeO**  =  optic tectum; Cb  =  Cerebellum; **LLv**  =  ventral part of the lateral lemniscus; **Slu**  =  nucleus semi lunaris. Scale bars in **A** and **C** = 400 µm, in **B** = 600 µm.

In all major groups of vertebrates there are retinofugal visual fibers projecting from the brain to the retina (for a complete review see [1] and [2]). Retinofugal visual fibers are particularly well developed in birds, as first described by Cajal [3], [4] and Dogiel [5]. In birds, the majority of the cells giving rise to the retinofugal fibres are found in the isthmo optic nucleus (ION), a group of cells in the most dorso-caudal part of the isthmal region of the midbrain (see Fig. 1B–D; [6]–[9]).

Despite a large number of anatomical, physiological and histochemical studies, the function of the retinofugal system in birds remains unclear and a wide range of hypotheses have been proposed (reviewed in [1] and [10]). Some suggest the ION is involved in selective shifting of visual attention in the retina, either between relevant stimuli [11]–[15] or between the ventral and dorsal parts of the retina [10], [16], [17]. Alternative hypotheses include: involvement in the saccadic suppression of retinal activity [18], [19] enhancement of peripheral vision [20] and modulation of temporal processing [21]. In addition, the more complex organization and larger number of cells of the ION in pecking birds (and the smaller size in non-pecking birds) has led to the hypothesis that the ION is involved in ground feeding, either visually searching for small objects or in the control of pecking behavior [7], [22]–[25].

In vertebrates, sensory specializations are often correlated with increases in the size of brain areas associated with that specialization (“The principle of proper mass”, [26]). This has been shown repeatedly among vertebrates in relation to not only sensory specializations, but also motor skills and ‘complex’ behaviors (e.g. [27]–[31]). In most of these studies, the correlation between a structure and a behavior is established with an *a priori* knowledge that the structure is related to the generation of the behavior or sensory modality. In the case of the ION, the opposite strategy has been applied; the relative size and organization of the structure has driven some of the theories about its’ function. Although the ‘ground–feeding hypothesis’ is congruent with the published comparative data, the ION has only been described for a few orders and cell numbers are available for even fewer species. If comparative data is to be used to aid in determining the function of the retinofugal system, then a broad comparative analysis of the relative size and organization of the ION, comprising a diversity of bird species with different ecological niches and feeding habits, is required. In this study, we compared the cytoarchitectonic organization and relative volume of the ION in 81 species of birds belonging to 17 different orders to gain further insight on the function and evolution of the retinofugal pathway in birds. Because previous studies (e.g. [7], [23], [32], [33]) focused on cell counts as an indicator of the ION size, we also counted the number of cells in 58 of these species to examine the number and density of cells in the ION among birds.

Using this broad, comparative dataset, we tested two of the theories regarding ION function. First, if the ground-feeding hypothesis is correct, we would expect that all ground-feeding birds, regardless of what order they belong to, will have enlarged ION volumes relative to brain volume and that the ION will contain a greater number of cells. Conversely, species that do not feed on the ground, such as hummingbirds, parrots and some songbirds and pigeons, are expected to have relatively smaller IONs with fewer cells. Second, Wilson and Lindstrom (2011; [10]) recently proposed that the ION is involved in the detection of aerial predators and predicted that the ION should be enlarged and have more cells in birds that are heavily predated upon by other birds. From a comparative perspective, we would predict that parrots, coots, pigeons, some songbirds, galliforms (i.e. quail, pheasant and relatives) and waterfowl, which are under significant predation pressure from aerial predators [34]–[36], would have enlarged IONs containing more cells. The corollary of this theory is that groups that are rarely predated by other birds, namely owls, diurnal raptors (i.e., hawks and falcons), woodpeckers, nightjars and seabirds, should have relatively small IONs with fewer cells. Given the reported diversity of cell numbers and size of the ION among some avian orders, we also examined changes in the relative size and morphology of the ION across several phylogenetic trees to assess how the ION has evolved.

## Materials and Methods

### Ethics Statement

All specimens were provided to us dead by conservation authorities, wildlife veterinarians and museum staff and thus approval was not required by an institutional ethics committee to undertake this research.

### Measurements

We measured the relative volume, number of cells and cytoarchitecture of the ION in 83 specimens representing 81 species (table S1). In addition we examined the gross cytoarchitecture of several additional specimens loaned to us from museums. For these museum specimens, the volume and number of cells of the ION were not measured because of potential tissue shrinkage arising from long term storage of museum specimens in 70% ethanol. A complete list of these museum specimens is provided in table S2.

While we did report the cytoarchitectonic organization of the ION in the domestic chicken (*Gallus domesticus;* see results, Fig. S1) we did not include this species in our volumetric or cells number analyzes because we have concerns relating to the domesticated nature of this species. Several studies (e.g [37]–[39]) have shown that domestication has profound effects on the relative size of different parts of the brain, as well as in the overall brain size, both in birds and other vertebrates.

For all specimens in which the ION volume was measured, the head was immersion-fixed in 4% paraformaldehyde in 0.1 M phosphate buffer. The brain was then extracted, weighed to the nearest milligram, cryoprotected in 30% sucrose in phosphate buffer, embedded in gelatin and sectioned in the coronal or sagittal plane on a freezing stage microtome at a thickness of 40 µm. Sections were collected in 0.1 M phosphate buffered saline, mounted onto gelatinized slides, stained with thionin and coverslipped with Permount. The olfactory bulbs were intact in all of the specimens that we collected and sectioned. All brains were cut following bird brain atlases (e.g. [40], [41]) in which the brainstem ends at the same rostrocaudal point as the cerebellum. In this manner, brain measurements were consistent among our specimens.

Photomicrographs of every second section were taken throughout the rostrocaudal extent of each nucleus using a Retiga EXi *FAST* Cooled mono 12-bit camera (Qimaging, Burnaby, BC, Canada) and OPENLAB Imaging system (Improvision, Lexington, MA, USA) attached to a compound light microscope (Leica DMRE, Richmond Hill, ON, Canada). Exceptions to this were four owl species in which photomicrographs were taken of every fourth section because the remaining series were required for an unrelated study. Measurements of all the nuclei were taken directly from these photos with ImageJ (NIH, Bethesda, MD, USA; http://rsb.info.nih.gov/ij/) and volumes were calculated by multiplying the area in each section by the thickness of the section (40 µm) and the sampling interval. For those species represented by more than one specimen (table S1), the average of the measurements was taken as the species’ given value.

### Borders of Nuclei

The ION lies in the dorsal isthmus, medial to the caudo-dorsomedial edge of the optic tectum, at the level of the trochlear nucleus (Fig. 1A–D). Because no previous studies have described in detail the morphology and cytoarchitecture of the ION in a large groups of birds we provide detailed descriptions below. Briefly, the ION consists of a darkly stained group of cells that lies lateral and posterior to the root of the mesencephalic trigeminal nerve and medial to the parvocellular part of the nucleus isthmi, at the same level as the trochlear nucleus (Fig. 1 A–D).

### Cells Counts

We counted the number of cells in the ION in 59 species for comparison with previous studies [7], [23], [32], [33], [42], [43]. In several specimens, cells counts were not obtained. Although the Nissl stain was of sufficient quality to establish the borders of the ION, it did not allow us to differentiate between the cell nuclei and nucleoli, and therefore precluded an accurate estimation of cell numbers.

Cells were counted in the same sections used for volume estimation using an unbiased stereological method, the optical fractionator [44], [45]. An unbiased counting frame [46] was positioned on the coordinates of a square lattice randomly superimposed on the section. Because of the large variation in absolute volume of the ION among the sampled species, both the size of the counting frame and the distance between the coordinates of the lattice were varied to assure a minimum count of 80 cells. The area of the counting frame was either 0.00118 mm^2^ or 0.003 mm^2^, while the distance between the coordinates was between 0.2 and 0.1 mm along each axis. At each sampling point, the thickness of the sections was determined as the distance between that of the first particle coming into focus and the last particle going out of focus [44]. An unbiased brick-counting rule [47], [48] was used. That is, an unbiased counting frame was projected onto the thickness of the section resulting in a cuboid with the upper, top and left planes as acceptable surfaces and all others as unacceptable surfaces. Thus, if a cell contacted the lower, bottom or right planes, it was not counted. The height of the counting brick was two thirds of the total measured thickness. Nuclear profiles containing a nucleolus were counted using a 100X objective. At least 80 cells were counted per ION across all specimens. Coefficients of error were calculated using Scheaffer’s estimator [49], [50] for non-homogeneous distributions of cells.

### Statistical Analyses

To examine scaling relationships, we plotted the log10-transformed volume of each brain region against the log10-transformed brain volume minus the volume of each specific region [51]. Allometric equations were calculated using linear least squares regressions using: (1) species as independent data points, and (2) phylogenetic generalized least squares (PGLS) to account for phylogenetic relatedness [52], [53]. We applied two models of evolutionary change as implemented in the MATLAB program Regressionv2.m (available from T. Garland, Jr., on request; [54], [55]): Brownian motion (phylogenetic generalized least-squares or PGLS) and Ornstein–Uhlenbeck (OU) [55], [56]. Akaike Information Criterion (AIC) was used to determine which model best fit the data. The model with the lowest AIC is considered to be the best fit [55]. Models with AIC different by less than 2 units can also be considered as having substantial support [57], [58]. Because different phylogenetic trees can yield different results [59], we tested four models based on the trees provided in Cracraft et al., (2004; [60]), Livezey and Zusi (2007; [61]), Davis (2008; [62]), and Hackett et al. (2008; [63]). Resolution within each order was provided by order- and family-specific studies [64]–[73], although this left several nodes unresolved. Phylogenetic trees, character matrices and phylogenetic variance-covariance matrices were constructed using Mequite/PDAP:PDTREE software [74], [75] and the PDAP software package (available from T. Garland upon request). Because the phylogeny was constructed from multiple sources, branch lengths were all set at 1, which provided adequately standardized branch lengths when checked using the procedures outlined in Garland et al. (1992; [76]). Unresolved nodes were treated as soft polytomies, with branch lengths between internal nodes set to zero [77]. Allometric equations based on standard statistics, and the PGLS and OU models, calculated for each of the four trees, were calculated for: (1) ION volume against brain volume, (2) ION cell numbers against ION volume and (3) ION cell density against brain volume (table S3). We also included the avian orders and ION complexity categories (see results) as covariates in tree models to see if there is an effect of the orders or categories on the different variables. Currently there is no phylogenetically corrected pair wise comparison available and therefore Tukey HSD post hoc tests where only performed on non-phylogenetically corrected statistics. Because of the low number of species in some groups (e.g. woodpeckers), we also used the relative size of ION expressed as a percentage of the total brain volume in order to provide further comparisons between the different orders.

Non-phylogenetically corrected statistics and post-hoc tests were performed in the software JMP (JMP, Version 7. SAS Institute Inc., Cary, NC, 1989–2007). Additionally, we calculated phylogeny-corrected 95% prediction intervals using the PDAP module [74] of the Mesquite modular software package [75] to look for any significant outliers. To map the cytoarchitectonic organization of the ION on to an avian phylogeny, we constructed a phylogenetic tree of the orders used in this study (table S1) based on the phylogenetic relationships established by Hackett et al., (2008; [63]). While currently there is no consensus regarding the phylogenetic relationships among most orders of birds (e.g. [60]–[62], [78]), the use of different phylogenies in this part of the analysis did not alter our general conclusion and therefore we present only one of the possible phylogenies.

## Results

### ION Morphology

Because we observed great variation in the cytoarchitectonic organization of the ION among species, we developed a categorical grading system to quantify the degree of complexity of ION organization. The grading system consists of 6 numerical categories (0–5) that differ from one another in how much of the ION was organized into distinct layers (laminae). In species with less complex IONs, most cells are evenly distributed throughout the nucleus. As the complexity increases (see below), more cells are organized in layers and the amount of neuropil (cell-free lamina) increases.

#### Categrory 0

This category is characterized by the absence of a recognizable group of cells that can be identified as the ION. Species lacking an ION include the Chilean Tinamou (*Nothoprocta perdicaria*, Tinamiformes), seabirds (i.e., shearwater and albatross, Fig. 1A), the Australian Pelican (*Pelecanus conspicillatus* Pelecaniformes), and the Spotted Nightjar (*Eurostopodus argus,* Caprimulgiformes).

#### Category 1

**Figure 2:**
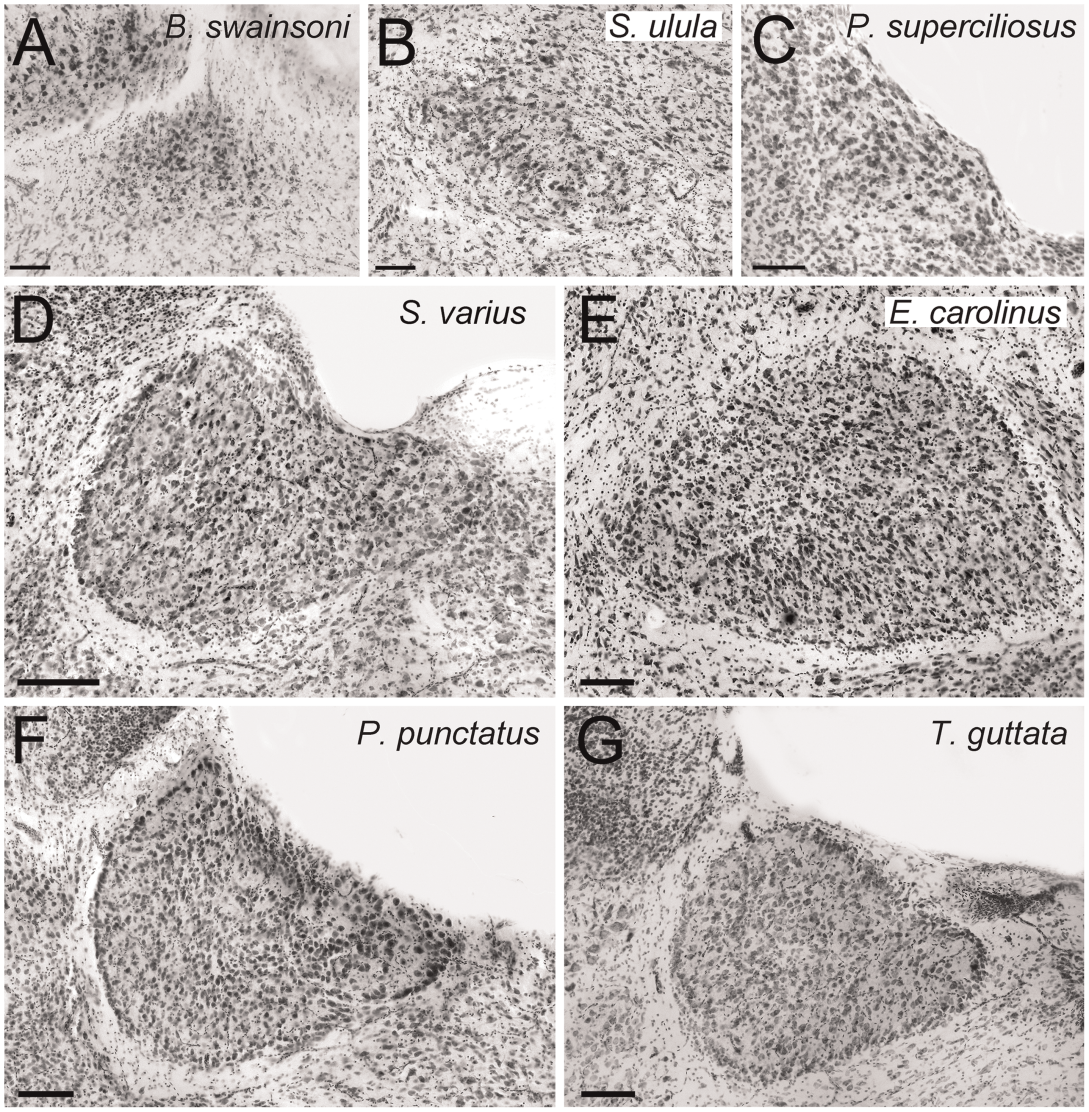
Variation of the complexity of the cytoarchitectonic organization of ION: Categories 1–3. Photomicrographs of coronal sections through the isthmo optic nucleus (ION) showing the variation of the complexity of the cytoarchitectonic organization of ION in different species of birds. **A** to **C** shows species that belong to category 1 of ION complexity (see methods). **A**, a diurnal raptor (Falconiformes), the Swainson’s Hawk (*Buteo swainsoni*); **B** an owl (Strigiformes), the Northern Hawk Owl, (*Surnia ulula*); **C** a hummingbird (Apodiformes), the Long-tailed Hermit (*Phaethornis superciliosus*); **D** and **E** shows species that belong to category 2 of ION complexity. **D** a woodpecker (Piciformes), the Yellow-bellied Sapsucker (*Sphyrapicus varius*); **E** a songbird **(**Passeriformes), the Rusty Blackbird (*Euphagus carolinus*); **F** and **G** shows species that belong to category 3 of ION complexity**.** **F** the Spotted Pardalote (*Pardalotus punctatus*); **G** the Zebra Finch (*Taeniopygia guttata*). **F** and **G** are both songbirds. Scale bars  = 100 µm.

In this category the ION is readily recognizable as an oval mass of evenly distributed cells. However, compared to other categories, the borders are somewhat indistinct (Fig. 2A–C).

#### Category 2

In species within category **2**, the border of ION is clearly defined and surrounded by a cell free neuropil (Fig. 2D, E). Most cells are evenly distributed throughout the nucleus, although the beginnings of lamination are present, insofar as there is a layer along the outer edge of the ION. However, this layer does not encapsulate the ION. (For example, see the lateral edge of ION in Fig. 2D and the medial edge in Fig. 2E).

#### Category 3

Compared to category **2**, ION in category **3** is characterized by a sharper border with a distinct layer of cells that encapsulates the rest of the nucleus (Fig. 2F, G). Also, in the category, there is a suggestion of neuropil adjacent to this exterior cell layer. Otherwise the cells are evenly distributed throughout the ION in a reticular manner (Fig. 2F, G).

#### Category 4

**Figure 3:**
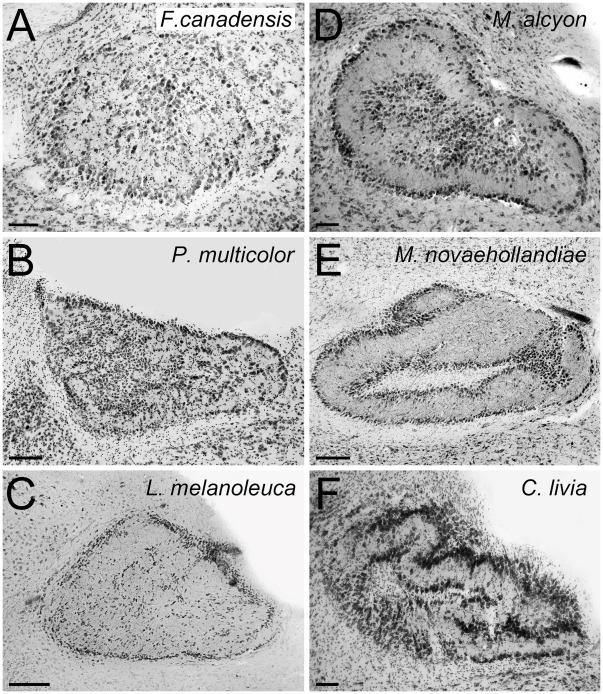
Variation of the complexity of the cytoarchitectonic organization of ION: Categories 4 and 5. Photomicrographs of coronal sections through the isthmo optic nucleus (ION) showing the variation of the complexity of the cytoarchitectonic organization of ION in different species of birds (see methods). **A** to **C** shows species that belong to category 4 of ION complexity. **A** shows a Galliform, the Spruce Grouse (*Falcipennis canadensis*); **B** showa songbird, the Scarlet Robin (*Petroica multicolor*); **C** shows a Columbiform, the Wonga Pigeon (*Leucosarcia melanoleuca*); **D** to **F** shows species that belong to category 5 of ION complexity. **D** a Coraciiform, the Belted Kingfisher (*Megaceryle alcyon*); **E** a songbird, the Superb Lyrebird (*Menura novaehollandiae*); **F** the Rock Pigeon (*Columba livia*). Scale bars = 100 µm, in **E** and **C** = 200 µm.

In category **4**, a neuropil is clearly recognizable within the external layer of cells. Nonetheless, some cells still are distributed in a non-laminated fashion within the ION (Fig. 3A–C).

#### Category 5

Finally, in category **5**, all cells appear to be organized into distinct layers both peripherally and within the ION, with a clearly recognizable neuropil between the layers of cells. Also, both the cell layers and the neuropil are thicker than in the other categories (Fig. 3D–F).

**Figure 4:**
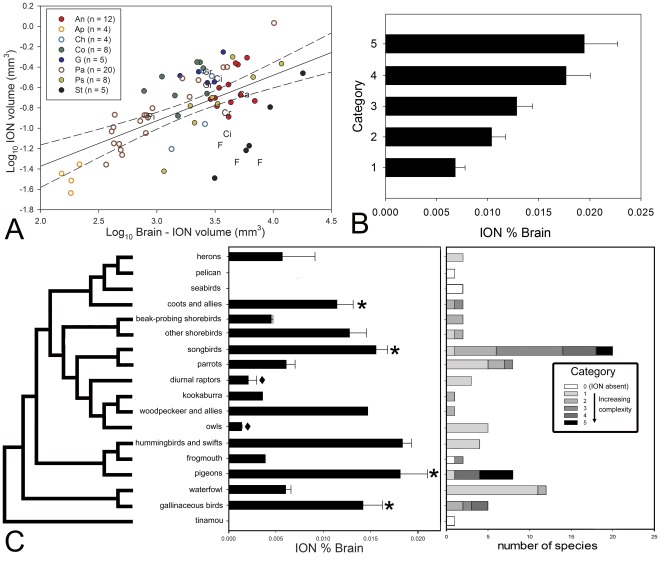
Variation of relative volume of ION and relation to ION complexity. **A**, Scatterplot of the isthmo optic nucleus (ION) volume plotted as a function of brain minus ION volume for all species examined (see table S1). **n** indicates to the number of species measured in each order. **B**, Bar graph of the relative size of ION expressed as a percentage of total brain volume grouped by the ION cytoarchitectonical complexity categories; the error bars indicate standard error. **C**. Phylogenetic relations among orders of birds surveyed in this study based on Hackett et al. (2008; 60]). The black bar graphs represent the relative size of ION expressed as a percentage of total brain volume for the different groups of birds. The error bars indicate standard error. The asterisk (*) indicates the groups in which a lower field myopia has been described [105]–[108]. The black diamond (♦) indicates species where a lack of lower field myopia has been described [109]. The colored bars represent the number of species that were examined of each ION cytoarchitectoncial organization complexity category in each order (see results, Table S1, Fig. 2). **An**  =  Anseriformes (red full circles); **Ap**  =  Apodiformes (empty orange circle); **Ca**  =  Caprimulgiforms; **Ch**  =  Charadriiforms (empty light blue circle); **Ci**  =  Ciconiiformes; **Co**  =  Columbiforms (dark green full circles); **Cr**  =  Coraciiforms; **F**  =  Falconiforms; **G**  =  Galliformes (dark blue full circle); **Gr**  =  Gruiformes; **Pa**  =  Passerifomes (empty brown circles); **Pi**  =  Piciforms; **Ps**  =  Psittaciformes (full yellow circle); **St**  =  Strigiforms (full black circle).

These categories are widely spread among orders. In all diurnal raptors (Fig. 2 A), owls (Fig. 2B), hummingbirds (Fig. 2C) and herons, the ION was classified as category **1**. Inspection of several other hummingbird species and two swift species from museums (see table S2) demonstrated that a simple cytoarchitectonical organization of the ION is widespread in the Apodiformes. In waterfowl, all species belong to category 1 except for the Lesser Scaup (*Aythya affinis*), which belongs to category 2. Among parrots, more than half of the species studied were classified as category 1 (5/8 species). The remaining three species were classified in category *2* (2 sp.) or 3 (table S1, Fig. 4C). Shorebirds also have a less complex ION, with all species in categories 1 and 2. Coots and allies appear to have moderately complex IONs with species in categories 2 and 3. Pigeons and doves also show relatively uniform complexity of their IONs, with almost all species in categories **4** and **5** (table S1, Fig. 3C, F, 4C). The exception is the Brush Bronzewing, *Phaps elegans*, (category **1**). Within the order Piciformes, the Yellow-Bellied Sapsucker (*Sphyrapicus varius*) has an ION in category 2 (Fig. 2D), but inspection of the museum specimens demonstrated that members of other families within the order have more complex IONs (table S2, Fig. 4C). A similarly diverse range of ION morphologies occurs in the galliforms, where ION complexity ranges from categories **2** to **4** (Fig. 3A, 4C). While not included in our volumetric analysis (see methods), inspection of a domestic chicken shows that this species has an ION in category 4 (Fig. S1). An even broader range occurs in the kingfisher (Coraciiformes), even though they were only represented by two species. The Laughing Kookaburra (*Dacelo novaeguineae*) has a less complex ION (category **2**) but the Belted Kingfisher (*Megaceryle alcyon*), has a very complex ION (category **5,** Fig. 3D). Finally, songbirds have the greatest variation in ION complexity of all of the orders that we examined, with species spanning categories **1** through **5** (Fig. 2E–G, 3 B, E, 4C).

### Relative Size of ION

ION varies greatly among taxa not only in morphology but also in relative size (Fig. 4). A regression of ION volume against brain volume with orders as a covariate shows a significant effect of order on the relative size of ION. Based on AIC values, the OU approach yields the best fit for all phylogenies and corroborates the significant effect of order on the relative size of ION in the four phylogenies used (table S3). Tukey HSD post hoc comparisons indicated that pigeons, galliforms and songbirds have significantly larger relative ION volumes than waterfowl, parrots, owls and diurnal raptors. Also, the woodpecker, hummingbirds, non beak-probing shorebirds, coots, waterfowl and parrots have significantly larger relative ION volumes than owls and diurnal raptors. Pigeons and hummingbirds, for example, have relative ION volumes (expressed as a percentage of the total brain volume) that are about three times that of parrots and waterfowl and nine times that of diurnal raptors and owls (Fig. 4C). Songbirds have, on average, IONs that are relatively smaller than those of pigeons and hummingbirds but which are still 8 times larger than those in diurnal raptors and owls. The Yellow Bellied Sapsucker (a woodpecker) has a relative ION volume between that of songbirds and galliforms. It is twice the size of parrots and waterfowl and more than 7 times that of diurnal raptors and owls. Also, galliforms and coots have IONs that are 5 to 6 times bigger than diurnal raptors and owls. Finally, beak-probing shorebirds, the frogmouth (a caprimulgiform) and the Laughing Kookaburra have relative small ION volumes, similar to diurnal raptors and owls.

Not only are there large differences among orders but also substantial variation within some orders. For example, among songbirds, the relative size of ION (expressed as a percentage of the total brain volume) in the Brown Thornbill (*Acanthiza lineata*, 0.031) is three times that of other songbirds, like the Superb Lyrebird (*Menura novaehollandiae*, 0.0106) or the Australian Magpie (*Cracticus tibicen*, 0.0099). Similarly, within the order Charadriiformes, there is a clear difference in the relative size of ION between beak-probing shorebirds and non beak-probing shorebirds (gulls) (Fig. 4A, C).

As shown in Figure 4A, there is considerable scatter around the regression line depicting the relationship between ION and brain size. The correlation coefficients associated with the regression lines derived from conventional statistics and the phylogenetically corrected statistics using both models of evolutionary change (PGLS and OU) are all below 0.5 (table S3), indicating that brain size explains less than 50% of the variation in ION size. Phylogeny-corrected prediction intervals showed that only the Swainson’s hawk (*Buteo swainsonii*) as an outlier and only when Davis’ (2008; [62]) phylogeny is used.

Not only does relative ION size vary among orders but also among our categories of cytoarchitectonic organization. Figure 4B shows the relative size of ION expressed as a percentage of the total brain volume grouped by ION complexity. Inclusion of ION categories as covariate in a regression shows that there is a significant effect of ION complexity on the relative volume of ION (table S3). The evolutionary model with the lowest AIC (the OU model) corroborates the significant effect of ION category on the relative size of ION for all phylogenies used (table S3). Tukey HSD post hoc comparisons showed that species scored as having a more complex ION (categories **4** and **5**) have relative ION volumes that are significantly larger than those in species scored as having category **1** IONs, but which are not significantly larger than those in species classified as having the others categories of ION. Although the other pair-wise comparisons are not statistically different, the general trend suggests that relative size of ION and its cytoarchitectonic organization is positively correlated (Fig. 4B). We also found that ION complexity is not related to either absolute ION volume or brain volume (data not shown).

We then mapped the distribution of ION relative size and complexity over one of the proposed phylogenies for birds (Fig. 4C; [63]). The results suggest that a relatively large ION has evolved independently several times, including: coots and allies, non beak-probing shorebirds, songbirds, woodpeckers, hummingbirds, pigeons and galliforms. Also our results show that a complex, laminated ION, with distinct cell layers and neuropil (categories **4**–**5,** Fig. 3) has evolved independently at least three times; in songbirds, pigeons and kingfishers (Fig. 4C). Our results also suggest that ION has been ‘lost’ at least two times independently, in the nightjar (Caprimulgiformes) and in the clade that includes the pelican and seabirds (Fig. 4C).

### ION Cells Numbers and Cell Density

**Figure 5:**
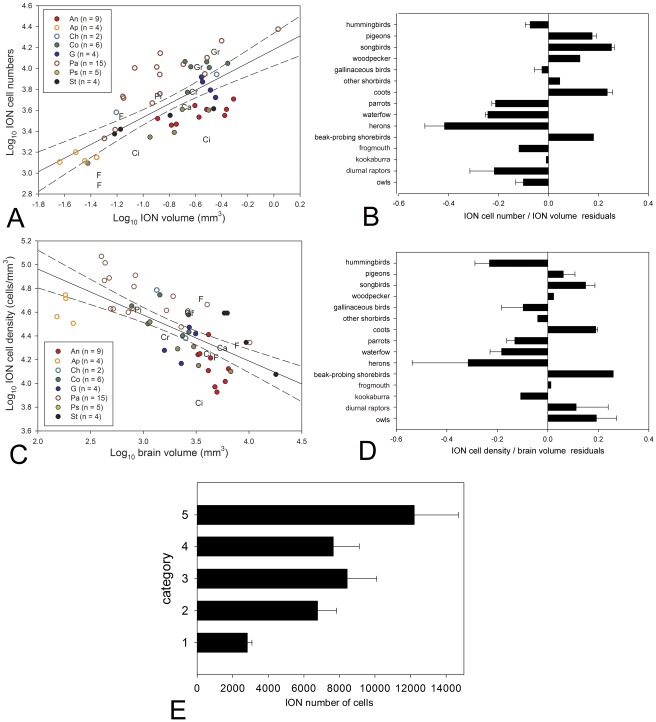
ION cells numbers and cells density variation among birds. **A**, Scatterplot of the cell numbers of ION plotted as a function of ION volume for all species examined (see table S1). The **n** between parentheses indicates the number of species measured in each order. **B**, Bar graph of the residuals of ION cell numbers against the ION volume (**A**) for different groups of birds; the error bars indicate standard error. **C** Scatterplot of the cell density (cells/mm^3^) in the ION, plotted as a function of brain volume for all species examined (see table S1). **D**, Bar graph of the residuals of ION cell density against the brain volume (**C**) for different groups of birds; the error bars indicate standard error. **E**, Bar graph of ION absolute cells numbers grouped by the ION cytoarchitectonic complexity categories; the error bars indicate standard error. **n** indicates to the number of species measured in each order. **An**  =  Anseriformes (red full circles); **Ap**  =  Apodiformes (empty orange circle); **Ca**  =  Caprimulgiformes; **Ch**  =  Charadriiformes (empty light blue circle); **Ci**  =  Ciconiiformes; **Co**  =  Columbiformes (dark green full circles); **Cr**  =  Coraciiformes; **F**  =  Falconiformes; **G**  =  Galliformes (dark blue full circle); **Gr**  =  Gruiformes; **Pa**  =  Passerifomes (empty brown circles); **Pi**  =  Piciformes; **Ps**  =  Psittaciformes (full yellow circle); **St**  =  Strigiformes (full black circle).

Cell numbers in the ION varied between 953 (CE  = 0.0831) in the Swainson’s Hawk to 23,760 (CE  = 0.0808) in the Superb Lyrebird (table S1). The highest cell density was 117,439 cells/mm^3^ in the Spotted Pardalote (*Pardalotus punctatus*) and the lowest cell density was 8,448 cells/mm^3^ in the Pacific Black Duck (*Anas superciliosa*; table S1). There is a significant positive correlation between ION cell numbers and ION absolute volume (Fig. 5A), but this explains only between 50 and 60% of the variation in cell number (table S3). The inclusion of orders as a covariate yielded a significant effect of group on cell number for both conventional statistics and the evolutionary model with the lowest AIC (OU) (table S3). Tukey HSD post hoc comparisons demonstrated that songbirds have significantly more cells in the ION than hummingbirds, waterfowl, parrots, herons, diurnal raptors and owls (Fig. 5B), after accounting for the size of the ION. Cell density (# cells/mm^3^) in the ION is also negatively correlated with the logarithm of brain volume (Fig. 5C; table S3). Thus, cell numbers increase with the absolute size of ION but cell density decreases with absolute brain size. A regression of ION cell density against the brain volume with order as a covariate revealed a significant effect of order on cell density for both conventional statistics and the OU evolutionary model (table S3). Tukey HSD post hoc comparisons show that songbirds and owls have a significantly higher cell density in the ION than hummingbirds, waterfowl, parrots and herons, relative to brain size (Fig. 5D). We found no effect of ION categories on the ION cell numbers relative to ION volume or cell density (data not shown).

A one way ANOVA yielded a significant effect of ION complexity categories on the absolute number of cells (table S3; Fig. 5E). The OU evolutionary model also shows a significant effect of ION complexity categories on the absolute number of cells (table S3). Tukey HSD post hoc comparisons showed that birds in category **1** have significantly fewer cells than birds in all other categories of ION complexity and species in category **5** have significantly more cells than species in category **2**. We also found that the number of cells in the ION, relative to ION volume, varies significantly among categories (ANOVA, F_4,53_ = 13.63, p<0.001; Fig. 5F). Tukey HSD post hoc comparisons indicated that species in category **1** have significantly fewer cells relative to ION volume than all other categories.

## Discussion

This is the first major systematic, comparative analysis of relative size, cytoarchitecture and cell number in the ION, the principal origin of retinofugal fibres in birds. The present study expands greatly the number of orders and species in which ION is described and provides a broad phylogenetic base onto which functional hypothesis can be tested and revised.

### ION Cytoarchitecture

In several species we found a lack of a recognizable assemblage of cells that could be classified as the ION (table S1). This is not the first time an ‘absence’ of the ION has been reported in birds. For example, the Brown Kiwi (*Apteryx australis*; [79]), the Wood Stork (*Mycteria americana*; [80]), and the Ostrich (*Struthio camelus*; [81]) all reportedly lack a recognizable ION. It is not clear whether ION is truly absent in these species or whether the ION is just extremely small. Unfortunately it is not known whether there are isthmal cells that project to the retina in any of these species, which might indicate the presence of an ION. Crocodilians, the living vertebrates most closely related to birds [82], do have centrifugal projections to the retina from cells in the isthmal region [83]–[85]. However, these cells are more similar to the ectopic cells of birds and there is no evidence that crocodilians have an ION [85]. In birds, ectopic cells also project to the retina but to different targets and are thought to have a different function from that of cells in ION (see [1], [10]). Interestingly, the Ostrich and the Kiwi, along with the Chilean Tinamou, all belong to the most ancestral group of birds, Paleognathae [63]. The absence of a recognizable ION in Paleognathae and the crocodilians suggests that the ION may have evolved first in the more modern group of birds, Neognathae, and that Paleognathae are more similar to Crocodilians with only ectopic cells that project to the retina.

Mapping the differences in cytoarchitectonic organization and relative size of ION on top of an avian phylogeny (Fig. 4C) reveals a complex pattern and suggests that evolutionary changes in both the relative size and the cytoarchitectonic organization of the ION have occurred independent of phylogeny. For example, Figure 4C shows that an ION with obvious cell layers and neuropils (Fig. 3, categories **4**, **5**) has evolved independently at least 4 times. In addition, birds with a relatively large ION tend to have a more complex ION (Fig. 4B), and birds with less complex ION organization (i.e., category **1**) have both fewer cells in both absolute and relative terms (see results). This suggests that the independent evolution of a complex laminated ION is associated with an enlarged ION, but also that a simple, reticular organization may be associated with fewer cells. This is well exemplified by hummingbirds; they have a large ION relative volume but have only around 1,000 cells and a simple ION organization (Fig. 4C). Thus, our data suggest that as ION increases in terms of relative size and the absolute number of cells, a more laminar organization is necessary to maintain or generate specific connections and/or firing properties.

The variation we observed in ION morphology is similar to the evolutionary transitions from a non-laminated to a laminated structure in other vertebrates (for a review see [86]). Examples of this include the dorsal lateral geniculate nucleus in mammals [87]–[89] and the vagal lobe of cyprinid fish [90]. Striedter (2005; [86]) proposed that one of the benefits of lamination is to reduce the length of neuronal connections thereby reducing transmission time and increasing processing power. In pigeons and galliforms, the dendrites of ION projection cells are directed towards the neuropil [91], [92] and both GABAergic interneurons and terminals from cells in the optic tectum, the main afferent of ION [92]–[95], lie exclusively in the neuropil [88]. This suggests that a more laminar organization of ION could be essential to maintain the interaction of tectal terminals, GABAergic interneurons and ION cells dendrites, which in turn would maintain the firing properties of ION cells. In fact, in quail, which have a laminated ION, the ION cells have large suppressive surrounds that include almost all of the remaining visual field and these properties depend exclusively on the GABAergic interneurons within the ION [15], [96]. Taken together this suggests that as the number of cells has increased in the ION, a more laminar organization has emerged to maintain the connections and response properties of ION cells.

### Relative Volume and Cell Numbers

Based on the total number of cells and morphology of ION, previous studies reported three types of ION: (1) songbirds, galliforms and pigeons have a well-developed, laminated ION, with around 10,000 cells [23], (2) waterfowl have a less differentiated, reticular ION with around 3,000 cells [23], [42] and (3) owls, diurnal raptors and birds that feed on the wing have a poorly developed ION with close to 1,000 cells [7], [23], [22], [33]. Based on a much larger number of species, our results confirm this pattern (Fig. 4A, C) and add several taxa. Hummingbirds, coots and non-beak probing shorebirds have relatively large IONs, similar to songbirds and pigeons. Parrots, beak-probing shorebirds, herons and the kookaburra have medium sized IONs, similar to waterfowl. Our results also confirm the very small IONs in owls and diurnal raptors (Fig. 4C). Cell numbers in ION in species that have been previously studied, like the rock pigeon (*Columba livia*, 9), the Common Blackbird (*Turdus merula*, [33]) and the mallard duck (*Anas platyrhynchos*, [42]) are very close to what we found. The one discrepancy is in the barn owl (*Tyto alba*), where Weidner et al. (1987; [7]) reported only 1,400 cells compared to the ≈2,500 we found. This discrepancy in the barn owl likely arises from a difference in counting methods because Weidner et al. (1987; [7]) did not use a rigorous stereological approach, as we did, to count cells.

Although we found a correlation between ION volume and ION cell numbers, this only explained about 50% of the variation in cell number (Fig. 5A). In agreement with this, we found significant differences in cell density among birds, after accounting for the influence of brain size on cell density (Fig. 5D). There seems to be a tendency for birds with large IONs to have higher cell density and birds with small IONs to have lower cell density, but this is not absolute. Among the groups with large IONs, galliforms and hummingbirds tend to have much lower relative cell densities. These differences in relative cell density may reflect differences in the functions or organization of the ION. For example, in galliforms the tectal projections to the ION arise from all parts of the tectum, whereas in pigeons, the projections largely arise from the ventral part of the tectum [91]–[94], [97]–[99]. It is possible that these differences influence the relative number of cells and volume of ION, but there is no information on tectal efferents to ION in any other groups of birds to test this hypothesis any further.

To date, most comparative studies of brain regions have focused on comparisons of relative volume (e.g. [31], [100]; but see [101]), but variations among species in the relative volume of a neural structure can be attributed to a variation (increases or decreases) in cell number and/or the amount and complexity of dendritic trees and terminals within the nucleus. Thus, comparing not only the relative volume, but also cell numbers and density can provide important clues on to how neural structures evolve. In the case of our study, both the relative volume and cell numbers of ION are associated with the cytoarchitectonic organization the nucleus and thus have provided insight in to the evolutionary transition from an unlaminated to a laminated structure.

### Control of Pecking Behavior

As mentioned before, the idea that the ION was larger in ground feeding and pecking birds (e.g., pigeons, songbirds and galliforms) and small in non-pecking birds (e.g., waterfowl, diurnal and nocturnal raptors) led various authors to propose that the ION is involved in the visual search of small objects or in the control of pecking behavior [7], [23]–[25], [32]. Although several groups adhere to this general distinction (like coots and non-beak-probing shorebirds; Fig. 4A–C), the data from other taxa casts doubt on the universality of this pattern. For example, the ION is relatively large in all of the songbirds and pigeons we measured, even though some species in these groups are not ground feeders, like the Eastern Spinebill (*Acanthorhynchus tenuirostris*), which is nectarivorous [102], or the Cedar Waxwing (*Bombycilla cedorum*) and the Torresian Imperial Pigeon (*Ducula spilorrhoa*) both of which feed largely on fruit in trees [103], [104]. This would at least indicate that ground feeding and searching for small objects is not the main driving force of the relative size of the ION. Similarly, hummingbirds have a relatively large ION (Fig. 4C) and they are highly specialized for feeding from flowers while hovering [105], which is not similar in any way to ground feeding or pecking.

### Aerial Predator Detection

Wilson and Lindstrom (2011; [10]) recently advanced the idea that the ION is involved in detecting images of shadows cast on the ground or on objects in the environment, which will then initiate a rapid and parallel search of the sky for a possible aerial predator. They based this on the anatomy and physiology of the retinofugal system, but also on the observation that ground-feeding birds have reportedly large IONs. Measuring the risk of aerial predation across the range of species we examined was not possible, but our results seem to be at odds with the aerial predation theory. For instance, we found that the ION is large and well developed in coots, which feed mostly in water bodies, and use similar nesting, feeding, brooding, and loafing sites as waterfowl (reviewed in [34]). The same aerial predators prey upon both coots and waterfowl, but these two groups have very different relative ION volumes (Fig. 4C). Also contradictory with Wilson and Lindstrom’s (2011; [10]) theory is the small size of ION in parrots. In most environments, parrots are subjected to predation by diurnal raptors (e.g. [35], [36]) and respond with alarm calls to the presence of such predators [106], so they should possess large rather than small IONs.

### New Hypothesis

Based on our observations of species differences in both size and morphology of ION, we propose an alternative theory for ION function. Several taxa that have both relatively large and complex IONs also have a lower field myopia (Fig. 4C). That is, asymmetries in the eye’s optical structure result in the dorsal part of the eye being myopic while the ventral part of the eye is emmetropic in these species, thereby keeping the ground in focus on the dorsal retina at the same time that the horizon and sky are in focus on the ventral retina [107]. Birds that have been described as having a lower field myopia include: pigeons [108], songbirds [109], galliforms [110] and coots [111], all which have relatively large IONs (Fig. 4C). Conversely, owls and diurnal raptors, both of which have small IONs, do not have a lower field myopia (Fig. 4C; [112]). Additional support for our hypothesis is provided by comparison the observations of Kolmer [113] on the optics of the Common Kingfisher (*Alcedo atthis*). In this species, the temporal retina is extremely myopic in air and only becomes emmetropic once it enters the water. Although we did not sample a Common Kingfisher, the Belted Kingfisher (Fig. 3D), which is also a diving species, has a complex, laminated ION. In contrast, the closely related Laughing Kookaburra has a simpler ION morphology that is relatively small (Fig. 4C) and is a terrestrial perch-hunting predator, similar to diurnal raptors in its foraging behavior and diet. Although we did not include the relative volume of ION in the Belted Kingfisher in our analyses because it is a museum specimen (see methods), the ION represented 0.015% of the total brain volume, which is much larger than that of the kookaburra and similar to that of galliforms and songbirds (Fig. 4C), suggesting that this species has an enlarged ION.

We therefore suggest that the ION is involved in switching attention between two parts of the retina i.e. from an emmetropic to a myopic part of the retina. In most cases this would mean switching from close range to long range vision in the retina. In the case of the birds with a lower field myopia this would be between the dorsal and ventral parts of the retina, but in the kingfisher this would be between the temporal retina and the rest of the retina. Birds with large IONs feed close to the substrate, which can include the ground, flowers and tree trunks and in species with a lower field myopia it is the part of the visual field containing the substrate that is myopic. On the other hand, birds with smaller IONs appear to feed far from the substrate, or have non-visually guided foraging behaviors. This is certainly true for galliforms, songbirds and pigeons that feed by pecking on the ground, but also true for species in these orders that feed mostly on fruit or insects in trees. Although both coot species that we examined feed in the water, they do so mostly by pecking, and while they sometimes submerge to feed [114], the small size of the beak and trigeminal system in these birds [100], suggests that they depend on vision for the detection of prey, probably at close range. The one woodpecker we examined also adheres to this pattern because it feeds on sap or insects on tree trunks at close range [115]. The difference in the relative size of the ION between beak probing and non-beak probing shorebirds is also in agreement with this hypothesis. Beak probing shorebirds feed close to the substrate, but they use tactile rather than visual cues to guide their foraging and their visual fields are adapted to attend to their surroundings and not the bill while foraging [116]. The situation in parrots is somewhat similar; parrots cannot see in the region below the bill and instead have more comprehensive visual coverage above the head [117]. The apparent ‘absence’ of the ION in the nightjar further supports our hypothesis as they feed by hunting insects in the air [118], which is in accordance with the previous reports that birds that feed on the wing have a reduced ION [33]. The reduced size of the ION in herons and the ‘absence’ of ION in seabirds and a pelican also fits our hypothesis; seabirds and pelicans usually dive into the water to catch fish, while herons have longs legs that keep them at a considerable distance from the ground when foraging [119]. Finally, that owls and diurnal raptors also have small and simple IONs is consistent with their feeding habits, which generally involve either perch hunting or feeding in the air [120].

Several studies have indicated that the effect of centrifugal fibers on nearby retinal ganglion cells is excitatory [14], [121]–[123]. This, in turn, suggests that the ION switches attention between different parts of the retina by increasing the responses of retinal ganglion cells. The anatomy of the centrifugal system provides good support to the idea of differential activation of parts of the retina as there is a clear asymmetry between the dorsal and ventral retina. In pigeons and galliforms, the ION projects exclusively to the ventral part of the retina (i.e. the dorsal visual field; [16], [124]–[126]. In the ventral retina, terminals from the ION make synapses with target amacrine cells (TCs), which project to both the ventral and the dorsal retina [16], [127]–[129]. Although the major synapses of the ION fibers in the retina are the TCs, there is evidence that some terminals from the ION synapse with targets other than TCs in the ventral retina [126], [130]. Therefore, the ION could enhance the responses of cells in the dorsal and ventral differentially; directly and through some TCs in the ventral retina, but only through TCs in the dorsal retina. Other authors [10], [17] have proposed that the ION is involved in switching attention between the dorsal and ventral retina for the primary purpose of predator detection. Our results do not support the notion that avian predator detection is the primary function of the ION, but predator detection would be one of the behaviors supported by the ability to switch attention between different parts of the visual field. While we believe that our hypothesis is general enough to explain the diversity of species with an enlarged ION, it is certainly true that the different hypotheses proposed so far are not mutually exclusive and that the ION could subserve different functions in different groups.

Although our new functional hypothesis is based on a much broader sampling of bird species, it requires experimental testing. For example, if our hypothesis is correct, then birds with relatively large IONs, like hummingbirds, woodpeckers and non beak-probing shorebirds, should be myopic parts in some parts of their visual fields and this myopia should match their respective feeding behaviors. Further, electrophysiological confirmation that projections from the ION alternatively activate parts of the retina that subserve the upper and lower (i.e. emmetropic and myopic) parts of the visual field in pigeons or galliforms will be necessary.

## Supporting Information

Figure S1 **Photomicrograph of a coronal section through the isthmo optic nucleus (ION) of a domestic chicken (** ***Gallus domesticus*** **). Scale bar = 100 µm.**
(TIF)

Table S1 **List of the Species Surveyed, Sample Sizes, Volumes (mm^3^), Number of Cells, Coefficients of Error (CE), and Cell Density (in cells/mm^3^) of the isthmo optic nucleus (ION).** Brain Volumes (mm^3^) for each species are also included.
(DOC)

Table S2 **List of species in which museum specimens where used to describe the cytoarchitecture of ION.**
(DOC)

Table S3 **Results of least-squares linear regression performed on the ION volume against brain volume, ION cell numbers against ION volume and ION cell density against brain volume are provided for ION using both species as independent data points (‘no phylogeny’) and two models of evolutionary change, Brownian motion (PGLS) and Ornstein-Uhlenbeck (OU;** [**52**] **,** [**53**] **) with four different phylogenetic trees.**
(DOC)
