# Supplementary figures and images for: Functional Implications of Species Differences in the Size and Morphology of the Isthmo Optic Nucleus (ION) in Birds
Source: PLoS One. 2012 May 29;7(5):e37816. doi: 10.1371/journal.pone.0037816 (PMC3362605; doi:10.1371/journal.pone.0037816)

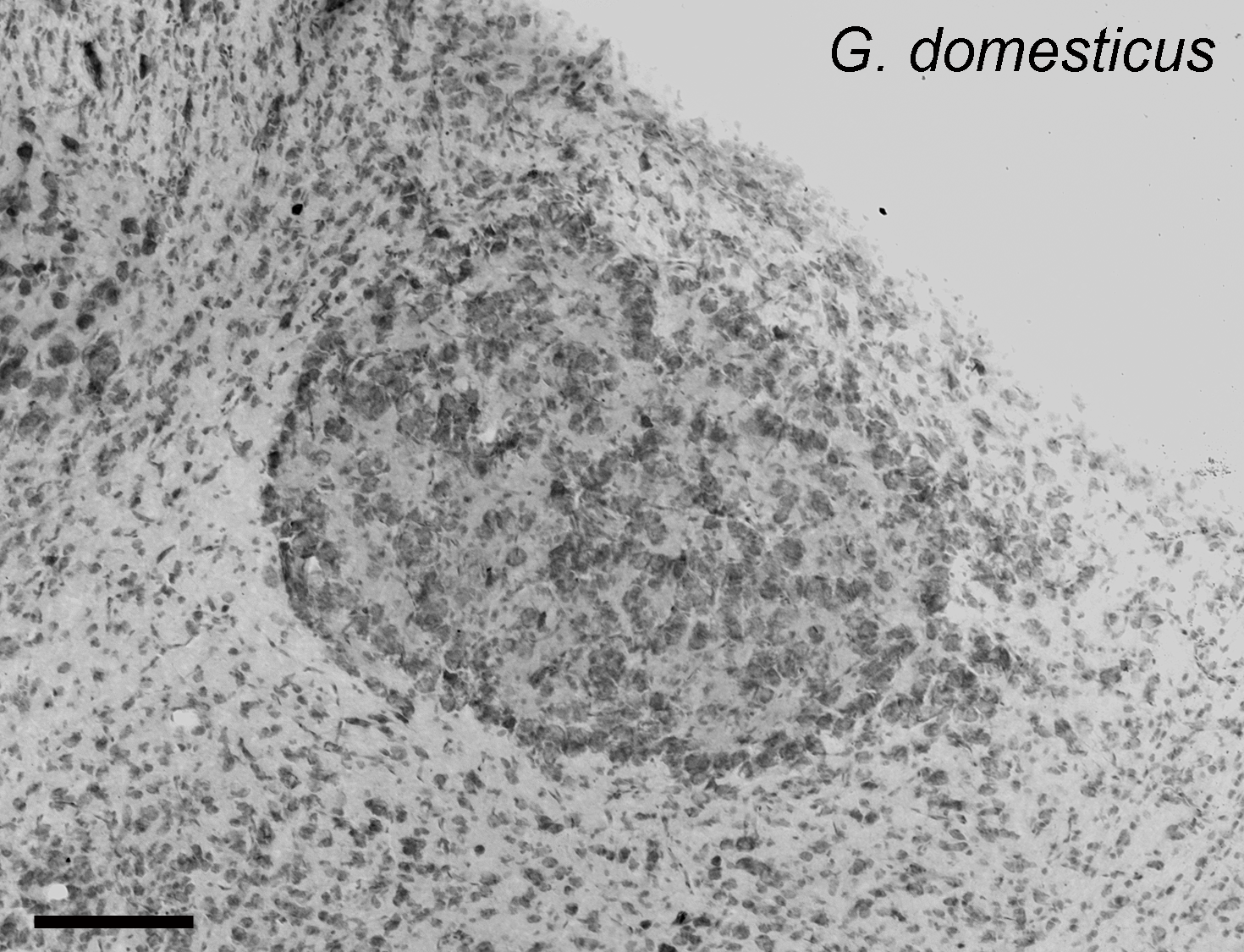

Supplement: Figure S1 — Photomicrograph of a coronal section through the isthmo optic nucleus (ION) of a domestic chicken ( Gallus domesticus ). Scale bar = 100 µm. (TIF) [file pone.0037816.s001.tif]
