# Supplementary material for: Functional Implications of Species Differences in the Size and Morphology of the Isthmo Optic Nucleus (ION) in Birds
Source: PLoS One. 2012 May 29;7(5):e37816. doi: 10.1371/journal.pone.0037816 (PMC3362605; doi:10.1371/journal.pone.0037816)
Supplement: Table S1 — List of the Species Surveyed, Sample Sizes, Volumes (mm3), Number of Cells, Coefficients of Error (CE), and Cell Density (in cells/mm3) of the isthmo optic nucleus (ION). Brain Volumes (mm3) for each species are also included. (DOC) [file pone.0037816.s002.doc]

**Table S1.** List of the Species Surveyed, Sample Sizes, Volumes (mm^3^), Number of Cells, Coefficients of Error (CE), and Cell Density (in cells/mm^3^) of the isthmo optic nucleus (ION). Brain Volumes (mm^3^) for each species are also included.

| **Order** | **Common name** | **Species** | **n** | **ION** | **Brain** | **ION N cells** | **CE** | **ION cell density** | **ION cytorchitecture** |
| --- | --- | --- | --- | --- | --- | --- | --- | --- | --- |
| Anseriformes | Green-winged Teal | *Anas carolinensis* | 1 | 0.198 | 3165.83 |  |  |  | 1 |
|  | Chestnut Teal | *Anas castanea* | 1 | 0.249 | 3424.71 | 4422.703 | 0.1018 | 17776.14 | 1 |
|  | Northern Shoveller | *Anas clypeata* | 1 | 0.165 | 3288.51 | 2873.016 | 0.0676 | 17450.29 | 1 |
|  | Blue-winged Teal | *Anas discors* | 1 | 0.193 | 2895.75 |  |  |  | 1 |
|  | Mallard duck | *Anas platyrhynchos* | 1 | 0.185 | 6949.81 | 4057.627 | 0.0930 | 13264.55 | 1 |
|  | Pacific Black Duck | *Anas superciliosa* | 1 | 0.420 | 4973.94 | 3552.301 | 0.0779 | 8448.206 | 1 |
|  | Lesser Scaup | *Aythya affinis* | 1 | 0.129 | 4141.89 | 3323.478 | 0.0672 | 25739.45 | 2 |
|  | Redhead | *Aythya americana* | 1 | 0.212 | 5245.17 |  |  |  | 1 |
|  | Bufflehead | *Bucephala albeola* | 1 | 0.268 | 4122.97 | 3427.739 | 0.0899 | 12805.36 | 1 |
|  | Common Goldeneye | *Bucephala clangula* | 1 | 0.492 | 5961.39 | 5102.61 | 0.1217 | 10377.91 | 1 |
|  | Australian Wood Duck | *Chenonetta jubata* | 1 | 0.180 | 4329.15 | 2934.413 | 0.1045 | 16345.88 | 1 |
|  | Red-breasted Merganser | *Mergus serrator* | 1 | 0.434 | 4754.34 | 4064.046 | 0.0741 | 9358.986 | 1 |
| Caprimulgiformes | Spotted Nightjar | *Eurostopodus argus* | 1 |  | 1013 |  |  |  | 0 (absent) |
|  | Tawny Frogmouth | *Podargus strigoides* | 1 | 0.214 | 5585.91 | 4264.234 | 0.0621 | 19889.15 | 3 |
| Charadriiformes | Silver Gull | *Chroicocephalus novaehollandiae* | 1 | 0.324 | 2968.15 |  |  |  | 1 |
|  | Bonaparte's Gull | *Chroicocephalus philadelphia* | 1 | 0.365 | 2512.55 | 3811.152 | 0.0901 | 61076.15 | 2 |
|  | Short-billed Dowitcher | *Limnodromus griseus* | 1 | 0.062 | 1338.03 | 8771.799 | 0.0928 | 24024.43 | 2 |
|  | Eurasian Woodcock | *Scolopax rusticola* | 1 | 0.110 | 2593.63 |  |  |  | 2 |
| Ciconiiformes | Nankeen Night Heron | *Nycticorax caledonicus* | 1 | 0.305 | 3360.04 | 1550.763 | 0.0714 | 17686.62 | 1 |
|  | Cattle Egret | *Bubulcus ibis* | 1 | 0.088 | 4025.1 | 2085.959 | 0.0990 | 6836.519 | 1 |
| Columbiformes | White-headed Pigeon | *Columba leucomela* | 2 | 0.444 | 2355.21 | 11191.43 | 0.1104 | 25224.11 | 4 |
|  | Rock Pigeon | *Columba livia* | 1 | 0.388 | 2508.93 | 11574.51 | 0.1067 | 37795.57 | 5 |
|  | Torresian Imperial Pigeon | *Ducula spilorrhoa* | 1 | 0.218 | 2697.88 | 5909.324 | 0.1234 | 27077.18 | 4 |
|  | Bar-shouldered Dove | *Geopelia humeralis* | 1 | 0.321 | 1106.18 | 10199.52 | 0.1157 | 31809.87 | 5 |
|  | Peaceful Dove | *Geopelia placida* | 1 | 0.232 | 776.062 | 10377.11 | 0.1354 | 44821.65 | 5 |
|  | Wonga Pigeon | *Leucosarcia melanoleuca* | 1 | 0.445 | 2217 |  |  |  | 4 |
|  | Brush Bronzewing | *Phaps elegans* | 1 | 0.132 | 1517.37 |  |  |  | 1 |
|  | Spotted Dove | *Streptopelia chinensis* | 1 | 0.209 | 1430.5 | 11630.59 | 0.0600 | 55659.42 | 5 |
| Coraciiformes | Laughing Kookaburra | *Dacelo novaeguineae* | 1 | 0.141 | 3970.08 |  |  |  | 2 |
| Falconiformes | Collared Sparrowhawk | *Accipiter cirrocephalus* | 1 | 0.046 | 4875.48 | 761.5132 | 0.1271 | 16468.71 | 1 |
|  | Swainson's Hawk | *Buteo swainsoni* | 1 | 0.046 | 7694.02 | 953.0021 | 0.0831 | 20753.53 | 1 |
|  | Merlin | *Falco columbarius* | 1 | 0.068 | 3509.65 | 3482.408 | 0.0656 | 51211.88 | 1 |
| Galliformes | Chukar Partridge | *Alectoris chukar* | 1 | 0.358 | 2284.75 | 5273.065 | 0.1326 | 14725.94 | 2 |
|  | Ruffed Grouse | *Bonasa umbellus* | 1 | 0.285 | 3124.9 | 7450.48 | 0.1301 | 26175.1 | 2 |
|  | Spruce Grouse | *Falcipennis canadensis* | 1 | 0.279 | 2720 | 8287.376 | 0.1098 | 29665.58 | 4 |
|  | Grey Partridge | *Perdix perdix* | 1 | 0.327 | 1582.2 | 6206.134 | 0.1267 | 18976.68 | 4 |
|  | Common Pheasant | *Phasianus colchicus* | 1 | 0.559 | 3721.7 |  |  |  | 3 |
| Gruiformes | American Coot | *Fulica americana* | 1 | 0.357 | 2718.92 | 14372.66 | 0.0828 | 40246.03 | 3 |
|  | Dusky Moorhen | *Gallinula tenebrosa* | 1 | 0.265 | 2726.54 | 10295.78 | 0.0981 | 38904.85 | 2 |
| Passeriformes | Brown Thornbill | *Acanthiza pusilla* | 1 | 0.135 | 434.363 | 14002.28 | 0.0725 | 103567.2 | 4 |
|  | Eastern Spinebill | *Acanthorhynchus tenuirostris* | 1 | 0.061 | 489.382 | 2598.557 | 0.0867 | 42515.66 | 4 |
|  | Cedar Waxwing | *Bombycilla cedrorum* | 1 | 0.089 | 805.3 |  |  |  | 3 |
|  | White-throated Treecreeper | *Cormobates leucophaea* | 1 | 0.134 | 781.853 | 5719.855 | 0.1101 | 42609.17 | 5 |
|  | Australian Magpie | *Cracticus tibicen* | 1 | 0.398 | 4017.37 | 18393.58 | 0.0744 | 46168.62 | 3 |
|  | Painted Firetail | *Emblema pictum* | 1 | 0.044 | 366.795 |  |  |  | 2 |
|  | Eastern Yellow Robin | *Eopsaltria australis* | 1 | 0.127 | 838.803 | 10325.34 | 0.0988 | 81276.33 | 3 |
|  | Gouldian Finch | *Erythrura gouldiae* | 1 | 0.071 | 427.606 | 5208.527 | 0.0890 | 73483.73 | 2 |
|  | Rusty Blackbird | *Euphagus carolinus* | 1 | 0.308 | 1656.56 | 12682.39 | 0.0891 | 41155.22 | 2 |
|  | Magpie-lark | *Grallina cyanoleuca* | 1 | 0.396 | 3731.66 |  |  |  | 1 |
|  | White-plumed Honeyeater | *Lichenostomus penicillatus* | 1 | 0.157 | 916.988 |  |  |  | 2 |
|  | Noisy Miner | *Manorina melanocephala* | 1 | 0.296 | 2278.96 | 8871.08 | 0.1269 | 29921.34 | 3 |
|  | Superb Lyrebird | *Menura novaehollandiae* | 1 | 1.077 | 10163.1 | 23760.38 | 0.0808 | 22059.18 | 5 |
|  | Spotted Pardalote | *Pardalotus punctatus* | 2 | 0.102 | 424.228 | 10127.94 | 0.1097 | 117439 | 3 |
|  | Scarlet Robin | *Petroica multicolor* | 1 | 0.070 | 473.938 | 5398.523 | 0.1234 | 77209.99 | 4 |
|  | Black-capped Chickadee | *Poecile atricapillus* | 1 | 0.134 | 814.479 | 8759.452 | 0.1066 | 65330.04 | 3 |
|  | Diamond Firetail | *Stagonopleura guttata* | 1 | 0.117 | 720.077 | 4666.459 | 0.1061 | 39734.83 | 3 |
|  | Double-barred Finch | *Taeniopygia bichenovii* | 1 | 0.093 | 409.266 |  |  |  | 4 |
|  | Zebra Finch | *Taeniopygia guttata* | 1 | 0.055 | 502.51 | 2143.779 | 0.1373 | 42400.7 | 3 |
|  | Common Blackbird | *Turdus melura* | 1 | 0.203 | 1914.09 | 10972.73 | 0.1079 | 54084.82 | 2 |
| Pelecaniformes | Australian Pelican | *Pelecanus conspicillatus* | 1 |  | 22500 |  |  |  | 0 (absent) |
| Piciformes | Yellow-bellied Sapsucker | *Sphyrapicus varius* | 1 | 0.130 | 888.4 | 5414.347 | 0.1012 | 41572.08 | 2 |
| Procellariiformes | Short-tailed Shearwater | *Puffinus tenuirostris* | 1 |  | 4757.72 |  |  |  | 0 (absent) |
|  | Black-browed Albatross | *Thalassarche melanophrys* | 1 |  | 14129.3 |  |  |  | 0 (absent) |
| Psittaciformes | Australian King Parrot | *Alisterus scapularis* | 1 | 0.501 | 4478.76 |  |  |  | 1 |
|  | Long-billed Corella | *Cacatua tenuirostris* | 1 | 0.430 | 11778 |  |  |  | 1 |
|  | Galah | *Eolophus roseicapilla* | 1 | 0.317 | 6723.94 | 3993.969 | 0.1363 | 12594.5 | 1 |
|  | Purple-crowned Lorikeet | *Glossopsitta porphyrocephala* | 1 | 0.165 | 1939.19 |  |  |  | 2 |
|  | Budgerigar | *Melopsittacus undulatus* | 1 | 0.038 | 1151.54 | 1243.332 | 0.0759 | 32927.21 | 2 |
|  | Cockatiel | *Nymphicus hollandicus* | 1 | 0.113 | 2111 | 2207.132 | 0.0883 | 19539.05 | 1 |
|  | Superb Parrot | *Polytelis swainsonii* | 1 | 0.199 | 2996.14 | 4066.512 | 0.1047 | 20414.22 | 1 |
|  | Rainbow Lorikeet | *Trichoglossus haematodus* | 1 | 0.173 | 3333.98 | 2447.665 | 0.0943 | 14138.54 | 3 |
| Strigiformes | Northern Saw-whet Owl | *Aegolius acadicus* | 1 | 0.032 | 3142.86 |  |  |  | 1 |
|  | Short-eared Owl | *Asio flammeus* | 1 | 0.067 | 6221.04 | 2626.059 | 0.0949 | 39078.26 | 1 |
|  | Great Horned Owl | *Bubo virginianus* | 1 | 0.346 | 17994.2 | 4122.411 | 0.0700 | 11906.22 | 1 |
|  | Northern Hawk-Owl | *Surnia ulula* | 1 | 0.161 | 9408.3 | 3560.759 | 0.0943 | 22122.01 | 1 |
|  | Barn Owl | *Tyto alba* | 1 | 0.060 | 5849.81 | 2363.641 | 0.1137 | 39081.37 | 1 |
| Trochiliformes | Anna's Hummingbird | *Calypte anna* | 1 | 0.031 | 183.88 | 1273.757 | 0.1061 | 55284.58 | 1 |
|  | Long-tailed Hermit | *Phaethornis superciliosus* | 1 | 0.044 | 216.15 | 1585.969 | 0.0999 | 51896.88 | 1 |
|  | Rufous-tailed Hummingbird | *Amazilia tzacatl* | 1 | 0.023 | 182.19 | 1412.858 | 0.1065 | 31994.08 | 1 |
|  | Rufous Hummingbird | *Selasphorus rufus* | 1 | 0.036 | 151.641 | 1305.641 | 0.0971 | 36429.73 | 1 |
| Tinamiformes | Chilean Tinamou | *Nothoprocta perdicaria* | 1 |  |  |  |  |  | 0 |
