# Supplementary material for: Functional Implications of Species Differences in the Size and Morphology of the Isthmo Optic Nucleus (ION) in Birds
Source: PLoS One. 2012 May 29;7(5):e37816. doi: 10.1371/journal.pone.0037816 (PMC3362605; doi:10.1371/journal.pone.0037816)
Supplement: Table S2 — List of species in which museum specimens where used to describe the cytoarchitecture of ION. (DOC) [file pone.0037816.s003.doc]

**Table S2:** List of species in which museum specimens where used to describe the cytoarchitecture of ION**.**

| **Order** | **Common name** | **Species** | **ION cytorchitecture** | **Specimen number*** |
| --- | --- | --- | --- | --- |
| Apodiformes | Glossy Swiftlet | *Collocalia esculenta* | 1 | FMNH SEA132 |
|  | Pygmy Swiftlet | *Collocalia troglodytes* | 1 | FMNH SEA133 |
|  | Speckled hummingbird | *Adelomyia melanogenys* | 1 | LSUMZ 129494, 129491 |
|  | Green-fronted lancebill | *Doryfera ludoviciae* | 1 | FMNH 320498 |
|  | Magnificent hummingbird | *Eugenes fulgens* | 1 | LSUMZ64774 |
|  | Rufous-breasted hermit | *Glaucis hirsuta* | 1 | USNM 616825 |
|  | Giant hummingbird | *Patagona gigas* | 1 | LSUMZ 123075 |
|  | Green-backed firecrown | *Sephanoides sephanoides* | 1 | FMNH 316786, 316784 |
|  | Fork-tailed woodnymph | *Thalurania furcata* | 1 | LSUMZ 123339 |
| Coraciiformes | Belted Kingfisher | *Megaceryle alcyon* | 5 | USNM430744 |
| Piciformes | Scaly-throated Honeyguide | *Indicator variegatus* | 4 | USNM638140 |
|  | Yellow-rumped Tinkerbird*,* | *Pogoniulus bilineatus* | 3 | USNM632982 |
|  | Emerald Toucanet | *Aulacorhynchus prasinus* | 2 | USNM540590 |

*Specimen numbers refer to the following institutions: USNM, National Museum of Natural History (Washington, DC); FMNH, Field Museum of Natural History (Chicago, IL); and LSUMZ, Louisiana State University Museum of Natural Science (Baton Rouge, LA).
