# Supplementary material for: Functional Implications of Species Differences in the Size and Morphology of the Isthmo Optic Nucleus (ION) in Birds
Source: PLoS One. 2012 May 29;7(5):e37816. doi: 10.1371/journal.pone.0037816 (PMC3362605; doi:10.1371/journal.pone.0037816)
Supplement: Table S3 — Results of least-squares linear regression performed on the ION volume against brain volume, ION cell numbers against ION volume and ION cell density against brain volume are provided for ION using both species as independent data points (‘no phylogeny’) and two models of evolutionary change, Brownian motion (PGLS) and Ornstein-Uhlenbeck (OU; [52] , [53] ) with four different phylogenetic trees. (DOC) [file pone.0037816.s004.doc]

**Table S3:** Results of least-squares linear regression performed on the ION volume against brain volume, ION cell numbers against ION volume and ION cell density against brain volume are provided for ION using both species as independent data points (‘no phylogeny’) and two models of evolutionary change, Brownian motion (PGLS) and Ornstein-Uhlenbeck (OU; [52], [53]) with four different phylogenetic trees.

|  | **Model** | **Regression** | | | | | **Group Effect** | | | | **Category Effect** | | | |
| --- | --- | --- | --- | --- | --- | --- | --- | --- | --- | --- | --- | --- | --- | --- |
| **ION volume / brain volume** |  | **d .f.** | **F** | **slope** | **r^2^** | **AIC** | **d.f.** | **F** | **p** | **AIC** | **d.f.** | **F** | **p** | **AIC** |
| No phylogeny |  | 1, 74 | 31.83 | 0.425 | 0.301 | 35.27 | 13, 61 | 10.55 | < 0.0001 | -28.29 | 4, 71 | 5.91 | < 0.0001 | 21.51 |
| Cracraft et al., 2004 [57] | PGLS | 1, 74 | 39.95 | 0.581 | 0.350 | 14.72 | 13, 61 | 1.98 | >0.05 | 14.03 | 4, 71 | 1.27 | 0.288 | 17.38 |
|  | OU | 1, 74 | 39.23 | 0.553 | 0.346 | 13.06 | 13, 61 | 9.56 | < 0.0001 | -26.29 | 4, 71 | 3.93 | >0.05 | 11.731 |
| Davis, 2008 [59] | PGLS | 1, 74 | 53.42 | 0.629 | 0.419 | 4.77 | 13, 61 | 1.51 | 0.136 | 9.87 | 4, 71 | 0.96 | 0.429 | 8.67 |
|  | OU | 1, 74 | 51.96 | 0.607 | 0.412 | 4.03 | 13, 61 | 9.48 | < 0.0001 | -26.29 | 4, 71 | 3.44 | >0.05 | 5.38 |
| Livezey and Zusi, 2007 [58] | PGLS | 1, 74 | 46.66 | 0.638 | 0.386 | 4.32 | 13, 61 | 1.56 | 0.119 | 8.87 | 4, 71 | 0.49 | 0.737 | 10.19 |
|  | OU | 1, 74 | 45.47 | 0.612 | 0.381 | 3.29 | 13, 61 | 9.47 | < 0.0001 | -26.29 | 4, 71 | 3.10 | >0.05 | 6.02 |
| Hackett et al., 2008 [60] | PGLS | 1, 74 | 41.16 | 0.599 | 0.357 | 13.32 | 13, 61 | 2.16 | >0.05 | 10.48 | 4, 71 | 1.28 | 0.285 | 15.95 |
|  | OU | 1, 74 | 41.37 | 0.571 | 0.358 | 10.10 | 13, 61 | 9.53 | < 0.0001 | -26.29 | 4, 71 | 3.47 | >0.05 | 9.05 |
| **ION cell N/ ION vol** |  | **d.f.** | **F** | **slope** | **r^2^** | **AIC** | **d.f.** | **F** | **p** | **AIC** |  |  |  |  |
| No phylogeny |  | 2, 56 | 53.01 | 0.649 | 0.481 | 7.10 | 12, 44 | 12.01 | < 0.0001 | -53.16 |  |  |  |  |
| Cracraft et al., 2004 [57] | PGLS | 2.56 | 71.77 | 0.557 | 0.561 | -39.49 | 12, 44 | 1.80 | 0.076 | -29.99 |  |  |  |  |
|  | OU | 2, 56 | 73.28 | 0.568 | 0.566 | -38.04 | 12, 44 | 7.11 | < 0.0001 | -50.83 |  |  |  |  |
| Davis, 2008 [59] | PGLS | 2, 56 | 77.28 | 0.582 | 0.579 | -35.18 | 12, 44 | 1.16 | 0.336 | -27.83 |  |  |  |  |
|  | OU | 2, 56 | 77.40 | 0.587 | 0.581 | -33.80 | 12, 44 | 6.91 | < 0.0001 | -50.83 |  |  |  |  |
| Livezey and Zusi, 2007 [58] | PGLS | 2. 56 | 84.87 | 0.582 | 0.602 | -37.41 | 12, 44 | 0.73 | 0.708 | -24.48 |  |  |  |  |
|  | OU | 2, 56 | 84.39 | 0.583 | 0.601 | -35.64 | 12, 44 | 6.83 | < 0.0001 | -50.84 |  |  |  |  |
| Hackett et al., 2008 [60] | PGLS | 2, 56 | 79.74 | 0.597 | 0.587 | -32.39 | 12, 44 | 1.76 | 0.084 | -32.15 |  |  |  |  |
|  | OU | 2, 56 | 79.83 | 0.605 | 0.587 | -31.48 | 12, 44 | 3.31 | < 0.05 | -50.90 |  |  |  |  |
| **ION cell den/ brain vol** |  | **d.f.** | **F** | **slope** | **r^2^** | **AIC** | **d.f.** | F | P | **AIC** |  |  |  |  |
| No phylogeny |  | 2, 56 | 45.17 | -0.388 | 0.444 | -13.05 | 12, 44 | 5.74 | < 0.0001 | -44.92 |  |  |  |  |
| Cracraft et al., 2004 [57] | PGLS | 2.56 | 21.75 | -0.353 | 0.279 | -24.27 | 12, 44 | 1.46 | 0.173 | -17.76 |  |  |  |  |
|  | OU | 2, 56 | 24.97 | -0.344 | 0.308 | -29.27 | 12, 44 | 5.31 | < 0.0001 | -42.91 |  |  |  |  |
| Davis, 2008 [59] | PGLS | 2, 56 | 25.39 | -0.345 | 0.311 | -25.81 | 12, 44 | 0.87 | 0.576 | -15.59 |  |  |  |  |
|  | OU | 2, 56 | 29.05 | -0.353 | 0.342 | -28.89 | 12, 44 | 5.20 | < 0.0001 | -42.91 |  |  |  |  |
| Livezey and Zusi, 2007 [58] | PGLS | 2, 56 | 28.30 | -0.356 | 0.335 | -27.67 | 12, 44 | 0.61 | 0.817 | -12.59 |  |  |  |  |
|  | OU | 2, 56 | 31.87 | -0.363 | 0.362 | -29.70 | 12, 44 | 5.04 | < 0.0001 | -42.91 |  |  |  |  |
| Hackett et al., 2008 [60] | PGLS | 2, 56 | 24.24 | -0.351 | 0.302 | -24.30 | 12, 44 | 1.06 | 0.406 | -16.58 |  |  |  |  |
|  | OU | 2, 56 | 28.83 | -0.355 | 0.339 | -29.28 | 12, 44 | 4.57 | < 0.0001 | -42.91 |  |  |  |  |
| **Absolut ION cell numbers** |  | d.f | F | *p* | AIC |  |  |  |  |  |  |  |  |  |
| No phylogeny |  | 4, 53 | 10.81 | < 0.0001 | 1121.03 |  |  |  |  |  |  |  |  |  |
| Cracraft et al., 2004 [57] | PGLS | 4.53 | 3.49 | < 0.05 | 1132.27 |  |  |  |  |  |  |  |  |  |
|  | OU | 4, 53 | 7.71 | < 0.0001 | 1122.23 |  |  |  |  |  |  |  |  |  |
| Davis, 2008 [59] | PGLS | 4, 53 | 2.816151 | < 0.05 | 1133.1 |  |  |  |  |  |  |  |  |  |
|  | OU | 4, 53 | 7.28 | < 0.0001 | 1122.1 |  |  |  |  |  |  |  |  |  |
| Livezey and Zusi, 2007 [58] | PGLS | 4, 53 | 1.69 | 0.164 | 1116.12 |  |  |  |  |  |  |  |  |  |
|  | OU | 4, 53 | 7.46 | < 0.0001 | 1117.99 |  |  |  |  |  |  |  |  |  |
| Hackett et al., 2008 [60] | PGLS | 4, 53 | 3.49 | < 0.05 | 1134.18 |  |  |  |  |  |  |  |  |  |
|  | OU | 4, 53 | 7.53 | < 0.0001 | 1121.88 |  |  |  |  |  |  |  |  |  |
